# Gallic Acid‐Mediated Enhancement of Diazepam‐Induced Sedation via GABA_A_ Receptor Modulation: In Vivo and In Silico Evaluation

**DOI:** 10.1002/fsn3.71793

**Published:** 2026-06-02

**Authors:** Noshin Tasnim Yana, Anike Chakrabarty, Chinmoy Kumar Saha, Nikhat Jamal Siddiqi, Norah Alnazhan, Md. Sakib Al Hasan, Feroz Khan Nun, Imam Hossen Rakib, Md. Nasimul Haque Shipon, Emon Mia, Md Asaduzzaman Jony, Ali Mohamod Wasaf Hasan, Muhammad Torequl Islam, Azmat Ali Khan

**Affiliations:** ^1^ Department of Pharmacy Gopalganj Science and Technology University Gopalganj Bangladesh; ^2^ Department of Biotechnology, Faculty of Engineering and Science University of Greenwich Kent UK; ^3^ Department of Pharmaceutical Technology, Faculty of Pharmacy University of Asia Pacific Dhaka Bangladesh; ^4^ Department of Medical Surgical Nursing, College of Nursing King Saud University Riyadh Saudi Arabia; ^5^ Pharmaceutical Biotechnology Laboratory, Department of Pharmaceutical Chemistry, College of Pharmacy King Saud University Riyadh Saudi Arabia; ^6^ Department of Pharmacy Mawlana Bhashani Science and Technology University Tangail Bangladesh; ^7^ Department of Chemistry York College of the City University of New York New York New York USA; ^8^ Pharmacy Discipline Khulna University Khulna Bangladesh

**Keywords:** gallic acid, insomnia, molecular docking, pharmacokinetics, sedation

## Abstract

Insomnia is a disorder that involves dissatisfaction with sleep and difficulty falling or staying asleep. This study aims to explore the sedative activity of gallic acid (GA) on thiopental sodium (TS)‐induced sleep in mice. Intraperitoneal (i.p.) injections of GA (50, 100, and 200 mg/kg) and diazepam (DZP) (2 mg/kg) were given to *Swiss* albino mice individually or in combination. After administering TS, the onset and sleep duration were recorded. Additionally, GA's binding affinity (BA) with the GABA_A_ receptor (α1 and β2 subunits) was examined using molecular docking. Results revealed that GA significantly (*p* < 0.05) decreased onset and improved sleep duration in comparison to the control group in a dose‐dependent manner. The highest dose of GA (GA‐200) showed significant sedative activity with latency (1.77 ± 0.76 s) and sleep duration (182.28 ± 2.87 min). The combination of GA with DZP showed a synergistic sedative effect through decreasing latency and increasing sleep duration. In silico findings demonstrated that GA exhibited higher BA (−9.8 kcal/mol) against the GABA_A_ receptor (α1 and β2 subunits) compared to DZP (−8.4 kcal/mol). It also exhibited good pharmacokinetic characteristics and was less toxic than the DZP. Overall, our in vivo and in silico studies suggest that GA showed promising sedative activity. However, further investigation, including clinical trials, is needed to discover its medicinal potential.

## Graphical abstract

Gallic acid (GA) dose‐dependently reduced sleep latency and prolonged sleep duration in thiopental sodium‐induced mice, with synergistic effects when combined with diazepam. Molecular docking revealed GA's higher binding affinity (−9.8 kcal/mol) to GABA_A_ receptor (α1 and β2 subunits) than diazepam (−8.4 kcal/mol), suggesting GABAergic potential interaction as the mechanism underlying its sedative activity.

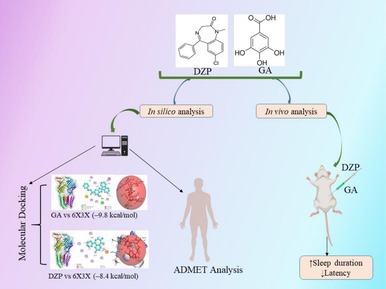

## 1 Introduction

Insomnia is a prevalent neurobehavioral disorder characterized by difficulty initiating or maintaining sleep, resulting in insufficient rest and impaired daytime functioning (Bos and Macedo 2019). Its prevalence increases with age, affecting approximately 20%–40% of the general population and up to 50% of individuals aged ≥ 65 years (Devi et al. 2018). Various factors are associated with insomnia, including stress (Yang et al. 2018), lifestyle behaviors, improper sleep schedules (Bos and Macedo 2019), and physical and mental health disorders (Sivertsen et al. 2021). Clinically, insomnia manifests as fatigue, reduced attention, and cognitive decline (Zhang et al. 2024). Consequences of insomnia include depression (Emamian et al. 2019), anxiety (Huang et al. 2020), hypertension (Cheng et al. 2015), tension‐type headache (Kim et al. 2017), impaired quality of life (Olfson et al. 2018), and increased risk of type 2 diabetes and incident heart failure (Hsu et al. 2015).

Sedation and hypnotic effects involve interaction with several neurotransmitter receptors, especially the GABAergic pathway (Brohan and Goudra 2017). GABAergic activity decreases neuronal excitability throughout the central nervous system (CNS), which results in sedation (Wang et al. 2017). GABA has two main types of receptors: GABA_A_ and GABA_B_ receptors. The GABA_A_ receptor acts mainly as an ion channel, allowing the flow of chloride ions into the neuron (Cherubini et al. 2022). In contrast, GABA_B_ receptors serve as G proteins to open potassium channels, resulting in potassium efflux and the production of inhibitory postsynaptic potentials (Karls and Mynlieff 2015). Almost all types of GABA_A_ receptors respond to benzodiazepines; only α4 or α6‐containing GABA_A_ receptors are immune to benzodiazepines (Crocetti and Guerrini 2020). According to several scientific reports, it has been demonstrated that the α1 and β2 subunits of GABA_A_ receptors are associated with sedative effects (Brohan and Goudra 2017). Also, abnormal functioning of the GABA_A_ receptor is responsible for anxiety (Nuss 2015), convulsions (Benarroch 2007), and Alzheimer's disease (Solas et al. 2015).

Sedative disorder are treated with various medications like benzodiazepines (Bisaga and Mariani 2015), barbiturates (Hay Kraus 2024), non‐benzodiazepine GABA agonists (Becker and Somiah 2015), antidepressants (Estrela et al. 2020), antihistamines (Krzystanek et al. 2020), serotonin antagonists (Monti 2011), and orexin receptor antagonists (Roecker et al. 2016) in order to improve relaxation, induce sleep, and lessen anxiety. However, DZP is the most popular benzodiazepine, with concerns about sedative, anxiolytic, anticonvulsant, and muscle relaxation properties (Murade et al. 2021). Although DZP is impactful, it leads to severe side effects such as acute kidney injury (Zhang et al. 2025), cardiac diseases (Al‐Abbasi et al. 2020), liver damage (Carvalhana et al. 2017), and respiratory depression (Singh et al. 2021). Also, long‐term use of DZP can cause physical dependency and lead to increased mortality (Edinoff et al. 2021). On the other hand, barbiturates like TS are associated with adverse effects: confusion, coma, ataxia, dysarthria, decreased mental status, and loss of brainstem reflexes (Suddock et al. 2024).

In order to cure insomnia, safer options are required because of these limitations. Since ancient times, natural products, especially medicinal plants, have been the basis for treating insomnia (Borrás et al. 2021). Bioactive compounds such as glucosides (Wei et al. 2017), flavonoids (Wang et al. 2023), tangeretin (Husain et al. 2024), polyphenols (Cho and Shimizu 2015), and terpenes (Wang et al. 2025) found in various medicinal plants would be alternative options for treating insomnia. For example, apigenin, a type of flavonoid chemical, is more successful in treating insomnia (Kramer and Johnson 2024). Also, several medicinal plants, including *Coriandrum sativum* L. (Apiaceae) (Santibáñez et al. 2023), *Crocus sativus* L. (Iridaceae) (Munirah et al. 2022), *Salvia leriifolia* Benth. (Lamiaceae) (Dobetsberger and Buchbauer 2011), *Schisandra chinensis* (Turcz.) Baill. (Magnoliaceae) (Liang et al. 2024), and *Mentha villosa* Huds. (Lamiaceae) (Motti and de Falco 2021), modulate the GABA_A_ receptor to treat insomnia and other sleep disruption disorders (Akram et al. 2019). Recent evidence also indicates that citronellal showed an excellent safety profile, interaction with the GABA_A_ receptor (α1 and β2 subunits), and substantial sedative effects both in vitro and in mice (Islam et al. 2025). However, people highly appreciate using natural products due to their minimal side effects, low cost compared to synthetic medication, and good therapeutic effects (Elkordy et al. 2021).

Gallic acid (GA) (3,4,5‐trihydroxybenzoic acid) is a crystalline solid available in various plants such as bearberry leaves ( *Arctostaphylos uva‐ursi* (L.) Spreng.) (Shamilov et al. 2021), green tea leaves ( *Camellia sinensis* (L.) Kuntze) (Karamać et al. 2006), grape seeds ( *Vitis vinifera* L.) (Karamać et al. 2006), evening primrose ( *Oenothera biennis* L.) (Timoszuk et al. 2018), and hazelnuts ( *Corylus avellana* L.) (Karamać et al. 2006). Numerous studies have reported that it exhibits a variety of bioactivities, such as anti‐inflammatory (Nguyen‐Ngo et al. 2020), anticancer (Jiang et al. 2022), anti‐hypoglycemic (Xu et al. 2021), antimicrobial (Choińska et al. 2021), antioxidant, antiviral (Hadidi et al. 2024), and antianxiety (Nagpal et al. 2013) properties. Additionally, GA strongly ameliorates several neurological disorders, including cardiovascular diseases (Kang et al. 2015), clot lysis and membrane stabilization (Sah et al. 2025), cerebral ischemia (Sun et al. 2014), Alzheimer's disease (Wahid et al. 2020), Parkinson's disease (Reckziegel et al. 2013), psychosis (Yadav et al. 2018), and neuropathic pain (Kaur and Muthuraman 2019). Consequently, GA showed neuroprotective effects, which suggests that it may enhance GABAergic regulation to produce sedative effects.

The aim of this study was to evaluate the sedative effects of GA using a TS‐induced sleep model in mice and to investigate its potential interaction with the GABA_A_ receptor through molecular docking and in silico pharmacokinetic analyses.

## 2 Materials and Methods

### 2.1 In Vivo Study

#### 2.1.1 Chemicals and Reagents

The experimental compound GA (CAS: 149‐91‐7, purity: ≥ 98.0% (HPLC)) was brought from Sigma‐Aldrich (USA). Loba Chemie Pvt. Ltd. (India) provided Tween‐80 (0.5%), and Square Pharmaceuticals Ltd. (Bangladesh) supplied DZP and TS.

#### 2.1.2 Dose Selection and Preparation

Previous literature suggests that GA displayed neurological effects in the experimental animals within 10 to 200 mg/kg doses (Hosseinzadeh et al. 2020; Mansouri et al. 2013). Therefore, test doses of 50, 100, and 200 mg/kg were selected for this investigation. A parent solution was prepared by adding a small amount of Tween‐80 (0.5%) and an adequate volume of distilled water (DW) to achieve a dose of 200 mg/kg. The parent solution was subsequently diluted to obtain doses of 100 and 50 mg/kg. DZP, used as the reference drug, was prepared at a dose of 2 mg/kg using DW. The treatment procedure and animal grouping are displayed in the study design sections (Table 1).

**TABLE 1:** Experimental groups and dosing regimen for the sedative activity evaluation of gallic acid and diazepam.

Treatment group	Description	Dose (mg/kg)
Control (Vehicle)	Distilled water containing 0.9% NaCl and 0.5% Tween 80	10 mL/kg
DZP‐2	Standard: Diazepam (GABA_A_ receptor agonist)	2
GA‐50	Lower dose	50
GA‐100	Middle dose	100
GA‐200	Higher dose	200
DZP‐2 + GA‐100	Standard + Test combination	2 + 100

*Note:* Control (vehicle): Distilled water containing 0.9% NaCl and 0.5% Tween 80; DZP: Diazepam 2 mg/kg; GA: Gallic acid; 50, 100, and 200 mg/kg; Every treatment is administered intraperitoneally (i.p.); (*n* = 7).

#### 2.1.3 Experimental Animals

Adult *Swiss* albino mice ( *Mus musculus* ; body weight 22–28 g) were purchased from the livestock section of Jahangirnagar University in Dhaka, Bangladesh. In the pharmacological lab at Gopalganj Science and Technology University (GSTU), these animals were kept in a controlled environment with a 12‐h darkness/daylight cycle at 24°C ± 3°C and 65% humidity. During this period, the animals had unrestricted availability of conventional meals and water. Before the sedative test, mice were starved for about 12 h. The current investigation took place between 9:00 a.m. and 3:00 p.m., and the mice were observed for 17 h to assess their potential mortality. The GSTU, Department of Pharmacy, approved the experimental design and methodology (#gstu‐21PHR006‐01).

#### 2.1.4 TS‐Induced Sleeping Test

The Turner (2013) methodologies were used to conduct the test. Following a few days of adjustment, 42 mice of both sexes were split into six groups at random, with seven mice in each group (*n* = 7) (Table 1). GA: 50, 100, and 200 mg/kg, DZP: 2 mg/kg, and control: 10 mL/kg were administered (i.p.). Each animal was administered TS (40 mg/kg, i.p.) to induce sleep 5 min before being placed in the observation chamber. Up to 4 h following the administration of TS, the length of sleep and the time until the loss of the righting reflex were recorded. The following formula was used to determine incidence of sleep.

$$\%\text{Incidence of sleep}=\left(\frac{\text{Number of slept mices}\;}{\text{Total mices in the group}}\right)\times 100$$

### 2.2 Statistical Analysis

All experimental data were analyzed using GraphPad Prism software (version 10.4.1, GraphPad Software, USA). Prior to statistical testing, the datasets were inspected for outliers and normal distribution. Data are presented as mean ± standard deviation (SD). The sample size (*n*) represents the number of independent observations in each experimental group as indicated in the corresponding figure legends. Statistical differences among multiple groups were evaluated using one‐way analysis of variance (ANOVA) followed by Tukey's honestly significant difference (HSD) post hoc test for multiple comparisons. A *p* value < 0.05 was considered statistically significant.

### 2.3 In Silico Study

#### 2.3.1 GABA Macromolecule Selection and Preparation

Two subunits (α1 and β2) of the GABA_A_ receptor (PDB ID: 6X3X) were selected from the RCSB Protein Data Bank based on their role in benzodiazepine‐induced sedation (Islam et al. 2025). Unnecessary components were removed from the protein structure using PyMOL software (v2.4.1) to optimize receptor performance and prevent docking interference. The SwissPDB Viewer tool was applied to energy minimization of receptor structure for the molecular docking process and storing the PDB file.

#### 2.3.2 Ligand Preparation

**FIGURE 1:**
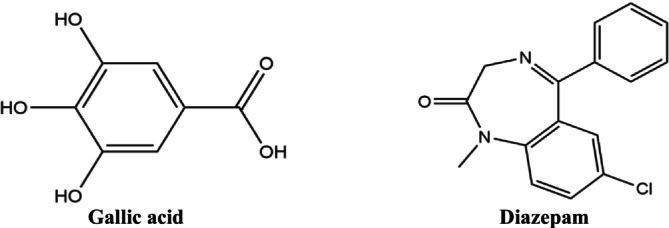
The 2D structures of gallic acid and diazepam.

The ligands 3D structures were downloaded in SDF format from the online database, PubChem (https://pubchem.ncbi.nlm.nih.gov/) to obtain docking findings. These include one standard medication, DZP (PubChem ID: 3016) and GA (PubChem ID: 370). The energy of ligands was reduced by using the MM2 (Allinger's force field) technique. Figure 1 displays the 2D structures of chosen ligands.

#### 2.3.3 Docking Protocol and Non‐Bond Interactions

The binding energies of the discovered ligands with the selected receptor were evaluated using PyRx. The PDB format of the ligand‐receptor complex was gathered and transformed into PDBQT format. Along the *x*, *y*, and *z* axes, a grid box of 35.02 × 39.37 × 25.00 was placed, and 200 steps were used to complete the docking calculation. The docking process's output was saved in CSV format. The software programs PyMOL (v2.4.1) and Discovery Studio (v21.1.020298) were used to visualize the connections between the ligand and protein. Furthermore, Bond lengths, hydrogen bonds (HB), and interacting amino acid residues were identified and analyzed.

#### 2.3.4 Pharmacokinetics, Drug‐Likeness Properties, and Toxicity

Pharmacokinetics and drug likeness properties in our investigation were predicted using the online tool SwissADME. Furthermore, the test drug's absorption, distribution, metabolism, excretion, and toxicity (ADMET) properties were evaluated using pkCSM, another web‐based tool (Omidkhah et al. 2023; Ahmad et al. 2023). Canonical SMILES of the compounds were obtained from PubChem (accessed March 19, 2025) and submitted to the ProTox‐3.0 platform to assess toxicity parameters.

## 3 Results

### 3.1 In Vivo Findings

In the TS‐induced sleep test, all animals exhibited loss of the righting reflex after TS administration, resulting in a 100% sleep incidence in all groups. Therefore, the evaluation of sedative activity was primarily based on sleep latency and sleep duration.

In vivo findings revealed that all treatment groups significantly decreased latency and increased sleep duration in comparison to the control group. The standard drug DZP‐2 group demonstrated a significant (*p* < 0.05) reduction in latency (1.31 ± 0.28 s) compared to the control group (3.34 ± 0.27 s). Similarly, the GA‐50 group showed a decrease in latency (2.48 ± 0.26 s) compared to the control group. The GA‐100 group showed a latency of 2.27 ± 0.53 s, which was less than that of the GA‐50 group and the control group's latency but higher than that of the DZP‐2 group and the GA‐200 group's latency. Furthermore, the GA‐200 group also exhibited decreased latency of 1.77 ± 0.76 s in comparison to the standard group DZP‐2 (1.31 ± 0.28 s) but showed lower latency compared to other individual treatments. Furthermore, the combination of GA‐100 and DZP‐2 resulted in the lowest latency (1.08 ± 0.12 s) among all treatment groups.

The control group showed a sleep duration of 141.66 ± 7.77 min. Administration of the DZP‐2 significantly (*p* < 0.05) increased sleep duration to 187.58 ± 4.16 min. Among the test compounds, GA‐100 demonstrated a sleep duration of 152.17 ± 2.11 min, which was higher than that of the control group (141.66 ± 7.77 min) and GA‐50 (150.06 ± 2.30 min) but lower than both DZP‐2 (187.58 ± 4.16 min) and GA‐200 (182.28 ± 2.87 min). Furthermore, the combination of GA‐100 with DZP‐2 resulted in the highest observed sleep duration of 188.14 ± 5.45 min among all treatment groups. Table 2 shows the mean value for each test sample.

**TABLE 2:** Effects of gallic acid and diazepam on sleep latency, duration, and incidence in thiopental sodium‐induced mice.

Treatments	Latency (s)	Duration of sleep (min)	Sleep incidence (%)
Control (Vehicle)	3.34 ± 0.27	141.66 ± 7.77	100
DZP‐2	1.31 ± 0.28*^bcd^	187.58 ± 4.16*^bcd^	100
GA‐50	2.48 ± 0.26*	150.06 ± 2.30*	100
GA‐100	2.27 ± 0.53*^b^	152.17 ± 2.11*^b^	100
GA‐200	1.77 ± 0.76*^bc^	182.28 ± 2.87*^bc^	100
DZP‐2 + GA‐100	1.08 ± 0.12*^abcd^	188.14 ± 5.45*^abcd^	100

*Note:* Values are mean ± SD (standard deviation) (*n* = 7); Statistical significance was determined using one‐way ANOVA followed by Tukey's post hoc test with multiple comparisons; *p <* 0.05 compared to the *Control (vehicle), ^a^DZP‐2, ^b^GA‐50, ^c^GA‐100, ^d^GA‐200.

Abbreviations: Control, distilled water containing 0.9% NaCl and 0.5% Tween 80; DZP‐2 + GA‐100, diazepam 2 mg/kg + gallic acid 100 mg/kg; DZP‐2, diazepam 2 mg/kg; GA‐100, gallic acid 100 mg/kg; GA‐200, gallic acid 200 mg/kg; GA‐50, gallic acid 50 mg/kg.

### 3.2 In Silico Findings

#### 3.2.1 Molecular Docking and Visualization of Ligand‐Receptor Interaction

**FIGURE 2:**
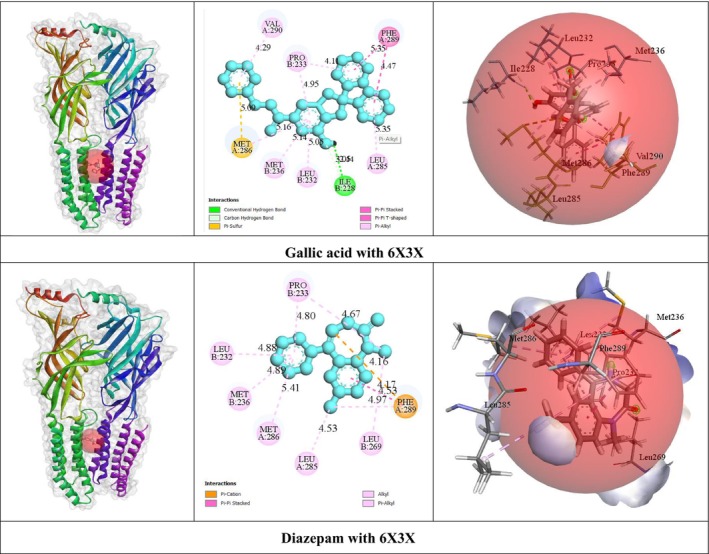
The two‐dimensional and three‐dimensional non‐bond interaction of gallic acid and diazepam with GABA_A_ receptor (α1 and β2 subunits).

In silico studies showed that GA exhibited the stronger BA (−9.8 kcal/mol) among GABA_A_ receptor (α1 and β2 subunits). However, GA did not form any HBs. On the other hand, it formed several HPBs, including MET A: 286, LEU A: 285, PHE A: 289, LEU B: 232, PRO B: 233 and VAL A: 290 with specific AA residues. Conversely, DZP displayed reliable BA (−8.4 kcal/mol) against GABA_A_ receptor, which was lower than GA. This interaction involved various HPBs: PHE A: 289, LEU A: 285, PRO B: 233, LEU B: 269, LEU B: 232, MET A: 286, and MET B: 236 AA residues. The number of HBs and 3D and 2D non‐bond interactions with the GABA_A_ receptor (α1 and β2 subunit types) were shown in Table 3 and Figure 2.

**TABLE 3:** Molecular docking results of diazepam and gallic acid towards the GABA_A_ receptor (α1 and β2 subunits), including BA, number of HB, and interacting amino acid residues.

Ligands	Receptors (PDB ID)	BA (kcal/mol)	No of HB	Amino acid (AA) residues	
				HBs	Others
DZP	GABA_A_ receptors (α1 and β2 subunits) (6X3X)	−8.4	—	—	PHE A: 289, LEU A: 285, PRO B: 233, LEU B: 269, MET A: 286, LEU B: 232, MET B: 236
GA		−9.8	—	—	MET A: 286, PHE A: 289, LEU A: 285, PRO B: 233, LEU B: 232, VAL A: 290

Abbreviations: AA, amino acid; BA, binding affinity; DZP, diazepam; GA, gallic acid; HB, hydrogen bond; PDB ID, Protein Data Bank identification code.

#### 3.2.2 Pharmacokinetics Properties, Drug‐Likeness, and Toxicity

In silico predictions show that GA is highly water‐soluble and satisfies Lipinski's Rule of Five, but these are theoretical values that need experimental validation. Unlike DZP, which is lipophilic and crosses the BBB by passive diffusion, GA is predicted to have poor BBB and CNS permeability (Log BB = −1.10; Log PS = −3.74). It has an excellent bioavailability score of 0.56. Also, GA has a molar refractivity (MR) of 39.47, which was within the acceptable range of MR ≤ 140. It showed limited intestinal absorption at 43.374% and significantly reduced Caco‐2 permeability at −2.56%. Furthermore, GA has no effect on substrates or inhibitors of CYP3A4, CYP2D6, or P‐glycoproteins I and II, with a total clearance of 0.518 log mL/min/kg, as shown in Table 4.

**TABLE 4:** Pharmacokinetics profile, toxicity and drug‐likeness properties of gallic acid.

Properties	Parameters	Gallic acid
Physicochemical properties	Formula	C_7_H_6_O_5_
	Molecular weight (g/mol)	170.12
	Number of aromatic heavy atoms	6
	Number of heavy atoms	12
	HBA	5
	HBD	4
	MR	39.47
Lipophilicity	Log P_o/w_ (XLOGP3)	0.70
Drug‐*likeness*	Lipinski	Yes; 0 violation
	Bioavailability score	0.56
Water solubility	Class	Very soluble
	Log S (ESOL)	−1.64
Absorption	Intestinal absorption (human) numeric (%absorbed)	43.374
	Caco2 permeability (log Papp in 10^−6^ cm/s)	−2.56
	Skin permeability (log Kp cm/h)	−2.735
	P‐glycoprotein I inhibitor	No
	P‐glycoprotein II inhibitor	No
Distribution	VDss (human) (log L/kg)	−1.855
	CNS permeability (log PS)	−3.74
	BBB permeability (log BB)	−1.102
Metabolism	CYP3A4 substrate	No
	CYP2D6 substrate	No
	CYP3A4 inhibitor	No
	CYP2D6 inhibitor	No
Excretion	Total clearance (log mL/min/kg)	0.518
	Renal OCT2 substrate	No
Toxicity	LD_50_ (mg/kg)	2000
	Toxicity class	4
	Carcinogenicity	Active
	Hepatotoxicity	Inactive
	Mutagenicity	Inactive
	Immunotoxicity	Inactive
	Cytotoxicity	Inactive

Abbreviations: BBB, blood brain barrier; CNS, central nervous system; HBA, hydrogen bond acceptor; HBD, hydrogen bond donor; H‐bond, hydrogen bond; LD_50_, lethal dose 50; MR, molar refractivity.

In our in silico toxicity study, GA is classified as a toxicity class 4 substance with a lethal dose of 50 (LD_50_) at 2000 mg/kg (Table 4). Although it has no adverse effects in case of hepatotoxicity, immunotoxicity, mutagenicity, or cytotoxicity, but showed predicted carcinogenic potential.

## 4 Discussion

The present study investigated the sedative potential of GA through both in vivo and in silico approaches, with particular emphasis on its interaction with the GABAergic system. Although benzodiazepines like DZP remain the mainstay of pharmacotherapy, their association with adverse effects including cognitive impairment, tolerance, dependence, and withdrawal symptoms necessitates the exploration of safer alternatives (Edinoff et al. 2021). Natural products have historically contributed to the development of CNS therapeutics, and GA, a polyphenolic compound widely distributed in medicinal plants including bearberry leaves ( *Arctostaphylos uva‐ursi* ), green tea ( *Camellia sinensis* ), and grape seeds ( *Vitis vinifera* ), has attracted attention for its neuropharmacological potential (Hadidi et al. 2024; Karamac et al. 2006; Shamilov et al. 2021).

The TS‐induced sleeping test serves as a well‐established experimental model for evaluating sedative‐hypnotic activity. TS, a barbiturate, potentiates GABAergic transmission by binding to the β‐subunit of the GABA_A_ receptor, thereby prolonging chloride channel opening and enhancing neuronal inhibition (Antkowiak and Rammes 2019; Yang et al. 2019). When GABA occupies its receptor site, TS extends the duration of chloride ion channel opening, allowing increased chloride influx that hyperpolarizes the neuronal membrane and reduces excitability (Mednikova et al. 2019; Krasowski and Hopfinger 2011). In this model, reduction in sleep latency and extension of sleep duration are indicative of CNS depressant activity. Our findings demonstrated that GA significantly reduced sleep latency and prolonged sleep duration in a dose‐dependent manner compared to the control group. The GA‐50 group showed reduced latency (2.48 ± 0.26 s) versus control (3.34 ± 0.27 s), while GA‐100 produced intermediate effects (2.27 ± 0.53 s), and GA‐200 exhibited the most pronounced effects among individual GA treatments, with latency of 1.77 ± 0.76 s and sleep duration of 182.28 ± 2.87 min. These results align with previous reports documenting the anxiolytic properties of GA in rodent models (Mansouri et al. 2014).

Notably, the combination of GA (100 mg/kg) with DZP (2 mg/kg) produced synergistic effects, yielding the shortest sleep latency (1.08 ± 0.12 s) and the longest sleep duration (188.14 ± 5.45 min) among all treatment groups. This observation suggests that GA may enhance benzodiazepine‐mediated GABAergic transmission, potentially through allosteric modulation or complementary mechanisms. Similar synergistic interactions have been reported for other polyphenolic compounds, including apigenin and tangeretin, which potentiate the effects of conventional GABAergic drugs while mitigating their toxicity profiles (Kramer and Johnson 2024; Husain et al. 2024). The combination strategy holds particular promise because it may allow for dose reduction of conventional sedatives, thereby minimizing their adverse effects while maintaining therapeutic benefit.

Accumulating evidence indicates that GA exerts CNS depressant, anxiolytic, and sleep‐modulatory effects potentially mediated through GABAergic signaling pathways. Mansouri et al. (2014) demonstrated that GA exhibits dose‐dependent anxiolytic and sedative effects, with its anxiolytic activity primarily mediated through 5‐HT_1A_ receptors rather than benzodiazepine binding sites, while higher doses additionally modulate GABAergic pathways to produce CNS depressant effects. The present study extends these observations by demonstrating sedative effects in a TS‐induced sleep model and providing computational evidence for direct GABA_A_ receptor interaction. Moreover, GA has been widely recognized for its diverse pharmacological properties, including antioxidant, cardioprotective, and neuroprotective effects (Uddin et al. 2022; Bhuia et al. 2023). These findings support the hypothesis that the observed behavioral effects may be associated with modulation of CNS pathways.

To elucidate the molecular basis of GA's sedative effects, we performed molecular docking studies targeting the GABA_A_ receptor α1 and β2 subunits (PDB ID: 6X3X). Molecular docking is a computational approach widely used in drug discovery to predict the preferred orientation and binding affinity of ligands when they interact with target proteins (Stanzione et al. 2021; Torres et al. 2019). Interestingly, GA exhibited a higher binding affinity (−9.8 kcal/mol) compared to DZP (−8.4 kcal/mol), despite lacking classical HB with the receptor. Both compounds shared several common interacting amino acid residues, including PHE A:289, LEU A:285, MET A:286, LEU B:232, and PRO B:233, suggesting comparable binding modes within the receptor's allosteric sites. The shared amino acid residues between DZP and GA indicate crucial binding sites that validate target engagement and support the test compound's efficacy (Lambrinidis et al. 2015). These findings are consistent with recent computational studies demonstrating that phenolic compounds can engage GABA_A_ receptors through hydrophobic interactions and π‐stacking, even in the absence of conventional hydrogen bonding (Islam et al. 2025). However, it should be acknowledged that binding energy predictions alone do not confirm functional activity, and electrophysiological studies are necessary to establish whether GA acts as a positive allosteric modulator, orthosteric agonist, or indirect modulator of GABAergic transmission.

In silico ADMET profiling is essential for understanding drug pharmacokinetics, efficacy, and safety during early drug development (Ruiz‐Garcia et al. 2008; Garcia‐Sosa et al. 2012). Our analysis revealed that GA complies with Lipinski's rule of five (zero violations), exhibits good bioavailability (0.56), and demonstrates moderate water solubility (Log *S* = −1.64, classified as very soluble). These properties are favorable for oral drug development. Molar refractivity (39.47) was within the acceptable range (≤ 140). However, GA showed limited predicted intestinal absorption (43.374%), reduced Caco‐2 permeability (−2.56 log Papp), and poor blood–brain barrier permeability (Log BB = −1.10; Log PS = −3.74), raising questions about its CNS bioavailability following systemic administration. This apparent discrepancy between predicted low CNS penetration and observed in vivo sedative effects may be explained by several factors: (1) active transport mechanisms not accounted for in computational predictions, (2) peripheral metabolites with enhanced CNS permeability, (3) accumulation in brain tissue despite slow permeation, or (4) indirect peripheral mechanisms that influence CNS function. Similar observations have been reported for other polyphenols, where poor predicted BBB permeability contrasts with documented CNS effects (Garcia‐Sosa et al. 2012). Future studies should include brain tissue distribution analysis to clarify this issue. GA showed no interaction with CYP3A4, CYP2D6, or P‐glycoproteins I and II, suggesting low potential for drug–drug interactions, with total clearance of 0.518 mL/min/kg.

**FIGURE 3:**
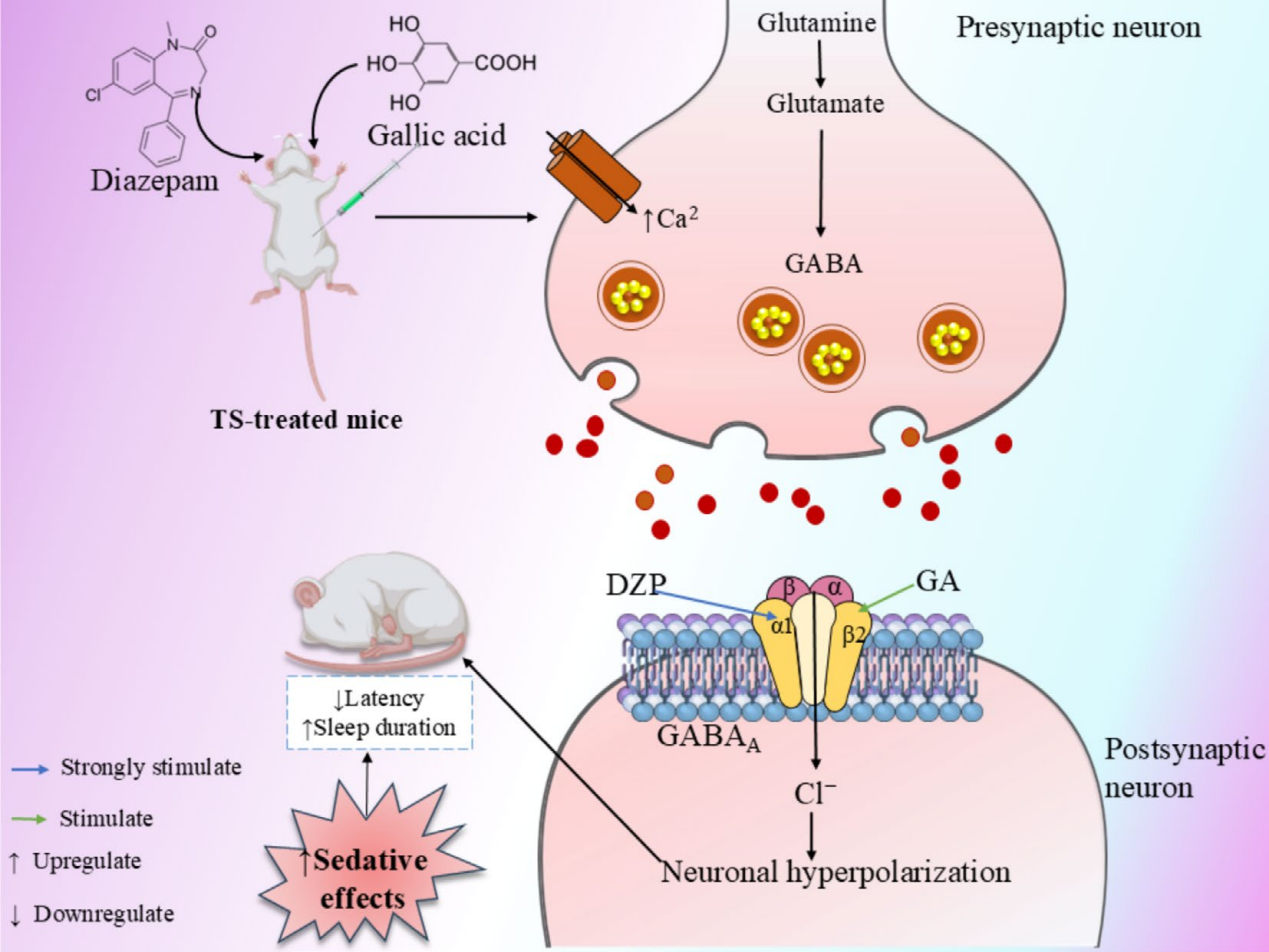
Possible mechanism of sedative activity of gallic acid. The proposed mechanistic pathway was adopted and inspired by previously published literature (Rakib et al. 2025) (Gallic acid [GA], when administered alone or with diazepam in TS‐treated mice, enhances GABA levels by increasing glutamate conversion to GABA in the presynaptic neuron, as shown in the diagram. It also boosts GABA_A_ receptor activity on the postsynaptic neuron, increasing chloride ion (Cl^−^) influx, which results in neuronal hyperpolarization. This hyperpolarization reduces neurological excitability, contributing to sedative effects and increased sleep duration, while decreasing latency).

Toxicological assessment using ProTox‐3.0 classified GA as toxicity class 4 with a LD_50_ of 2000 mg/kg, indicating relatively low acute toxicity (Szymański et al. 2011; Kramer et al. 2007). Importantly, GA showed no predicted hepatotoxicity, immunotoxicity, mutagenicity, or cytotoxicity, although carcinogenicity was flagged as active. This prediction warrants careful interpretation, as computational toxicity models may produce false positives, and confirmatory in vitro and in vivo genotoxicity studies are essential before clinical translation. Compared to DZP, which is associated with dependence, cognitive impairment, acute kidney injury (Zhang et al. 2025), cardiac effects (Al‐Abbasi et al. 2020), liver damage (Carvalhana et al. 2017), and respiratory depression (Singh et al. 2021), GA presents a potentially favorable safety profile pending experimental validation. Barbiturates like TS are associated with confusion, ataxia, dysarthria, and decreased mental status (Suddock et al. 2024), further supporting the need for safer alternatives. However, the possible mechanism of sedative activity of GA is shown in Figure 3.

However, several limitations must be acknowledged. First, the TS‐induced sleeping test, while widely used for preliminary screening, does not distinguish between direct GABAergic activation and indirect sedative effects mediated through other neurotransmitter systems. Second, the molecular docking analysis, although informative, requires validation through functional assays such as patch‐clamp electrophysiology or radioligand binding studies to confirm receptor engagement and characterize the nature of GA‐receptor interactions. Third, our study did not assess the effects of GA on sleep architecture using electroencephalography, which would provide more detailed insights into its hypnotic properties. Fourth, while reported binding energies provide comparative data, affinity constants were not calculated, which limits quantitative comparison of receptor occupancy (Lambrinidis et al. 2015).

In conclusion, this study provides convergent evidence from behavioral and computational analyses supporting GA's sedative potential, likely mediated through GABA_A_ receptor modulation. The synergistic interaction with DZP and favorable ADMET profile position GA as a promising lead compound for insomnia pharmacotherapy. However, the limitations inherent in predictive computational models and preliminary behavioral screening necessitate further investigation. Future directions should include: (1) electrophysiological characterization of GA effects on GABA_A_ receptor function using patch‐clamp techniques, (2) brain distribution and pharmacokinetic studies with quantitative analysis, (3) assessment of GA effects on sleep architecture using electroencephalography, (4) evaluation of chronic toxicity and genotoxicity through standardized assays, (5) exploration of structure–activity relationships through analog synthesis and testing, (6) mechanistic studies using selective antagonists to confirm receptor involvement, and (7) investigation of potential sex differences in response, as our study used both male and female mice but did not analyze results by sex. Such studies will establish whether GA can progress from a dietary polyphenol with promising preclinical activity to a clinically useful sedative agent for the management of insomnia and related sleep disorders.

## 5 Conclusion

In summary, GA has a considerable sedative effect by improving sleep duration and decreasing sleep latency. In our in vivo study, GA‐200 significantly (*p* < 0.05) decreased the sleep latency while increasing sleep duration in animals. Additionally, because GA‐100 has the lowest latency and the longest sleep duration, it works well with DZP‐2 to produce a sedative effect. Molecular study reveals that GA shows higher BA (−9.8 kcal/mol) to the GABA_A_ receptor (α1 and β2 subunits) in comparison to the standard drug DZP (−8.4 kcal/mol). GA's promise as a drug development candidate is supported by pharmacokinetic research, which shows that it maintains Lipinski's rule of five with an excellent bioavailability score as well as moderate water solubility. Toxicity studies also showed that GA had no adverse effect on many toxicities with an LD_50_ of 2000 mg/kg. Even though the study's findings suggest GA's promise as a sedative, future research should focus on electrophysiological validation of GABAergic mechanisms, brain distribution studies to resolve CNS permeability discrepancies, chronic toxicity assessment including carcinogenicity evaluation, and clinical trials to establish safety and efficacy in humans.

## Author Contributions

**Nikhat Jamal Siddiqi:** software, formal analysis, writing – review and editing. **Norah Alnazhan:** writing – review and editing, software, formal analysis. **Chinmoy Kumar Saha:** writing – original draft, methodology, investigation. **Azmat Ali Khan:** writing – review and editing, formal analysis, resources, funding acquisition. **Anike Chakrabarty:** writing – original draft, methodology, investigation. **Noshin Tasnim Yana:** conceptualization, writing – original draft, formal analysis, methodology, data curation. **Feroz Khan Nun:** software, formal analysis, writing – review and editing. **Md. Nasimul Haque Shipon:** visualization, software, formal analysis, investigation. **Muhammad Torequl Islam:** conceptualization, writing – review and editing, project administration, formal analysis, supervision, data curation, resources, validation. **Ali Mohamod Wasaf Hasan:** software, writing – review and editing. **Md Asaduzzaman Jony:** writing – review and editing, software. **Imam Hossen Rakib:** investigation, visualization, software, formal analysis. **Emon Mia:** investigation, visualization, formal analysis, software. **Md. Sakib Al Hasan:** conceptualization, writing – original draft, writing – review and editing, project administration, formal analysis, supervision, resources.

## Funding

The authors extend their sincere appreciation to the Ongoing Research Funding Program (ORF‐2025‐339) at King Saud University, Riyadh 11451, Saudi Arabia, for providing financial support for this research.

## Ethics Statement

The GSTU, Department of Pharmacy, approved the experimental design and methodology (#gstu‐21PHR006‐01).

## Conflicts of Interest

The authors declare no conflicts of interest.

## Data Availability

The data that support the findings of this study are available from the corresponding author upon reasonable request.
